# Smoke and Health

**Published:** 1923-06

**Authors:** 


### SMOKE AND HEALTH.

THE London County Council has just refused to urge the Minister of Health to introduce a Bill for the reduction of the volume of smoke which is the chief cause of the fogs for which the capital is notorious. The Council is as eagerly desirous as any public health authority ought to be for the removal of a nuisance which annually causes much inconvenience, serious danger, and a heavy death-roll, but it finds, or thinks it finds, itself in an administrative difficulty. It looks upon the Councils of the Metropolitan Boroughs as the proper authorities to deal with the matter, and it has therefore referred back the instructive report presented to it by the Departmental Committee which has recently dealt very thoroughly with the whole subject. It is to be feared therefore that, so far as London is concerned, legislation dealing with this subject is postponed indefinitely, since it is hopeless to expect that more than two dozen local authorities can be brought into line speedily or at all. The most important municipal body in the country is thus setting a very poor example to the others in a direction in which the pressure of public opinion is all important.

This decision in favour of masterly inactivity is the more to be regretted since the report of the Council's own committee contained abundant justification for immediate action. It is true that London fogs are much less frequent and severe than formerly, but it must not be forgotten that the mischief to health and the damage to property caused by black smoke continue daily and hourly and are by no means confined to foggy days. A comparatively brief reduction in the volume of smoke has a remarkable effect upon the death-rate from bronchitis. For the five years preceding 1921 the deaths from bronchitis in Scotland averaged 5,000 per annum—in 1922 they numbered 5,224. But in 1921, the year of the coal strike, they fell to 3,971—a tolerably clear indication that pure air and unclouded skies exercise an immediately beneficent effect upon the respiratory organs. What happens in London during periods of prolonged or frequently repeated fog we know only too well, and the progress that has already been made in clearing the air is a direct encouragement not merely to continue experiments which point the way to cleaner air and more sunlight, but to invoke the authority of the law to prevent the unnecessary pollution of the atmosphere. There is no mystery about the means of prevention, and the Public Control Committee of the London County Council enumerates many of them. More gas-cookers, better management of factory chimneys, the abolition of the old-fashioned kitchener—these are, perhaps, the most obvious means of purifying smoke-laden air. But let us not forget that there is now such a thing as smokeless fuel which, serving all the purposes of common coal, can be sold at the same price. The public at large know little of smokeless fuel, but the experts know, and they would substantially further their cause—which is everybody's cause—if they spoke more in its favour and less in favour of radiators, and other artificial—and detestable—methods of warming.

The experts have, in fact, stood very much in their own light, and, by their mistaken insistence upon unwelcome expedients, have deprived themselves of a good deal of support which might otherwise have been theirs. The average Englishman will continue a steady, persistent and justifiable opposition to all attempts to rob him of his cheerful open fire, and an "Act for the Abolition of Fireplaces and the Compulsory Use of Radiators" would not materially lessen that opposition. There is little to be gained by purifying the outer air if we make the atmosphere of our rooms stuffy and lifeless, and keep them at too high a temperature, as they invariably are when radiators are used. It is healthier to be rather cold than hot; but there is no reason that we should be either. A muggy air means a muggy-minded worker, and Englishmen, and more particularly Englishwomen, have no desire to acquire the dried up parchment complexions which result from life in an overheated atmosphere. To abolish the wasteful kitchener, which consumes much coal and produces a good deal of smoke, is one thing; to condemn the open fire, which, with reasonable attention to the teachings of science, need make practically no smoke at all, is a very different proposition, for which there will never be any enthusiasm in this country. We can surely diminish the nuisance and the danger of coal smoke without running counter to national sentiment. And it is national sentiment almost as much as national health and a clear atmosphere that are involved in this question.

